# Local DRLs and automated risk estimation in paediatric interventional cardiology

**DOI:** 10.1371/journal.pone.0220359

**Published:** 2019-07-31

**Authors:** Dimitri Buytaert, Kristof Vandekerckhove, Joseph Panzer, Lukas Rubbens, Daniël De Wolf, Klaus Bacher

**Affiliations:** 1 Department of Human Structure and Repair, Ghent University, Ghent, Belgium; 2 Department of Paediatric Cardiology, Ghent University Hospital, Ghent, Belgium; University of Nebraska Medical Center, UNITED STATES

## Abstract

**Introduction:**

Cardiac catheterization procedures result in high radiation doses and often multiple procedures are necessary for congenital heart disease patients. However, diagnostic reference levels (DRL) remain scarce. Our first goal was finding the optimal DRL parameter and determining appropriate DRLs. The second goal was to calculate organ doses (OD), effective doses (ED) and lifetime attributable risks (LAR) per procedure and to provide conversion factors based on dose area product (DAP).

**Materials and methods:**

DRLs are calculated for each procedure type, as the 75^th^ percentile of the cumulative value per procedure from the corresponding parameter. All irradiation events in the DICOM Structured Reports were automatically processed and simulated using PCXMC, resulting in OD, ED and LAR. Using a Kruskal Wallis H test and subsequent pairwise comparisons, differences in median values of the DRL parameter between procedure types were assessed.

**Results:**

Linear regression showed a strong correlation and narrow confidence interval between DAP and product of body weight and fluoroscopy time (BWxFT), even when all procedures (diagnostic and interventional) are combined. Only 15% of the pairwise comparisons were statistically significant for DAP normalized to BWxFT (DAP_BWxFT_). The latter pairs contained less frequent procedure types with significant outliers. For DAP normalized to BW (DAP_BW_), 38% of the pairwise comparisons showed statistically significant differences. Conversion factors from DAP_BW_ to OD and ED were reported for various weight groups, due to the higher correlation between DAP_BW_ and both OD and ED than between DAP and both OD and ED.

**Conclusions:**

The P75 of DAP_BWxFT_ for all procedures combined serves as an appropriate DRL value. This facilitates local DRL determination in smaller paediatric centres, which often have insufficient data to produce appropriate DRLs for different procedure types. Conversion factors are more reliable starting from DAP_BW_ instead of DAP and should be used according to the appropriate BW group.

## Introduction

The incidence of cardiac catheterizations in patients with congenital heart disease has increased over the last decade [1]. Due to improved technology, the procedures are becoming increasingly complex, resulting in high radiation doses delivered to the patient [2]. Complex therapies often need multiple interventions, thus increasing the cumulative dose to the patient [3]. Improved care significantly increased the life expectancy of the patients, increasing the time for stochastic radiation effects to develop. In addition, children are generally at higher lifetime radiation risk than adults and since they are smaller a larger fraction of their body is exposed to the direct X-ray beam [4–6].

Data on diagnostic reference levels (DRLs) of cardiac catheterization in congenital heart disease patients remain scarce. DRLs are simple threshold levels of easily measurable and available dose quantities, e.g. DAP in interventional cardiology, to identify examinations with unusually high patient doses [7]. Comparison of a department’s median exposure level to the DRL, can assist in identifying examinations with unusually high patient doses [8]. DRLs are therefore a valuable tool in optimizing and controlling radiation exposure to the patient. The PiDRL project by the European Society of Radiology (ESR), delivered European guidelines on DRLs for paediatric imaging in 2016. Recently these guidelines were published as RP 185 in the radiation protection series of the European Commission. In current study, the latter guidelines were applied to generate local DRLs.

Estimation of radiation risk to the population in cardiac catheterizations is most often done in literature by reporting effective dose. The organ and effective doses found in current literature are estimated by applying conversion factors from dose area product (DAP) values reported by the X-ray modality. Either a single conversion factor is applied to the cumulative DAP of a whole catheterization procedure, or several conversion factors are applied by performing Monte Carlo simulations or thermoluminescent dosimeter (TLD) measurements with anthropomorphic phantoms for a limited number of irradiation events per procedure and a limited variation in exposure parameters [6, 9–14]. In present study we calculated organ and effective doses by performing Monte Carlo simulations of all irradiation events (fluoroscopy and cinegraphy exposures) per procedure. We evaluated if the use of single conversion factors to calculate ED from DAP values (CF_ED_) is feasible.

Based on the simulated individual doses, lifetime attributable radiation risk (LAR) were calculated for each catheterization using the BEIR VII risk models. We determined appropriate organ dose conversion factors from DAP values from which the corresponding LAR can be calculated [5].

To our knowledge this is the first study taking into account all irradiation events and patient ages and sizes to automatically determine organ and effective doses and radiation risk and additionally to provide DAP to organ dose conversion factors (CF_OD_) appropriate for subsequent LAR determination.

## Methods and materials

### Imaging modality and data acquisition

All procedures were performed on a biplane Philips AlluraClarity FD20/10 cath lab (Philips Healthcare, Best, The Netherlands). The modality sends DICOM Radiation Dose Structured Reports (RDSR) at the end of each procedure to GE DoseWatch, a dose management software (GE Healthcare, Milwaukee, WI, USA). In DoseWatch, the exposure parameters (tube voltage and current, pulse width, number of pulses, additional filtration, DAP, air kerma, etc.), patient parameters (length, weight, sex, age) and geometric information (C-arm projection and angulation, source to image detector distance, table position, etc.) of any irradiation event and each procedure are easily accessible.

### Catheterization procedures

Data were anonymized and retrospectively obtained from 242 consecutive cardiac catheterizations performed in the paediatric interventional cardiology department at Ghent University Hospital. This study was approved by the Ethics Comittee of Ghent University Hospital (approval number 2016–1584). No written or oral consent was obtained from the patients since this was a retrospective study for which data were anonymized. The catheterizations were performed between January 2015 and September 2017. Procedures involving rotational angiography were excluded from the study since the RDSR did not contain sufficient information of these irradiation events to be able to perform Monte Carlo simulations. In the end, data of 222 procedures are remaining. Additional filtration of 1mm aluminum and 0.4mm copper was inserted for all exposures and anti-scatter grid was never removed. Procedures are grouped into diagnostic and interventional procedures. The latter are subdivided further into different procedure types, as recommended by RP 185 [7].

### Diagnostic reference levels

The European guidelines provide recommendations on how to establish and use DRLs for paediatric x-ray examinations and procedures. They recommend the use of air kerma-area product (here referred to as DAP) as the primary DRL quantity for interventional cardiology procedures. The 75^th^ percentile (P75) of the DAP distribution should be calculated for several recommended patient weight groups since paediatric patients (premature babies until obese adolescents) may vary in size by a factor of 200. They also suggest DAP normalized to body weight (DAP_BW_) as a useful parameter. In case DAP and body weight have a clear linear correlation, DAP_BW_ can be used as a single DRL parameter instead of determining different DRLs per weight group. Additionally, the product of body weight and fluoroscopy time (BWxFT) is briefly mentioned as a potentially useful parameter [7].

This study provides the 75^th^ percentile of DAP_BW_ distribution as a primary DRL parameter, for different procedure types. The usefulness of DAP normalized by BWxFT as the primary DRL parameter is assessed. The correlation between DAP and body weight on the one hand and between DAP and the product of fluoroscopy time and patient weight on the other hand, are investigated. Likewise, the 75^th^ percentile of the air kerma at interventional reference point, fluoroscopy time and number of cinegraphic frames are serving as additional DRL quantities as recommended. These data are retrospectively available in DoseWatch.

### Organ and effective doses

**Fig 1:**
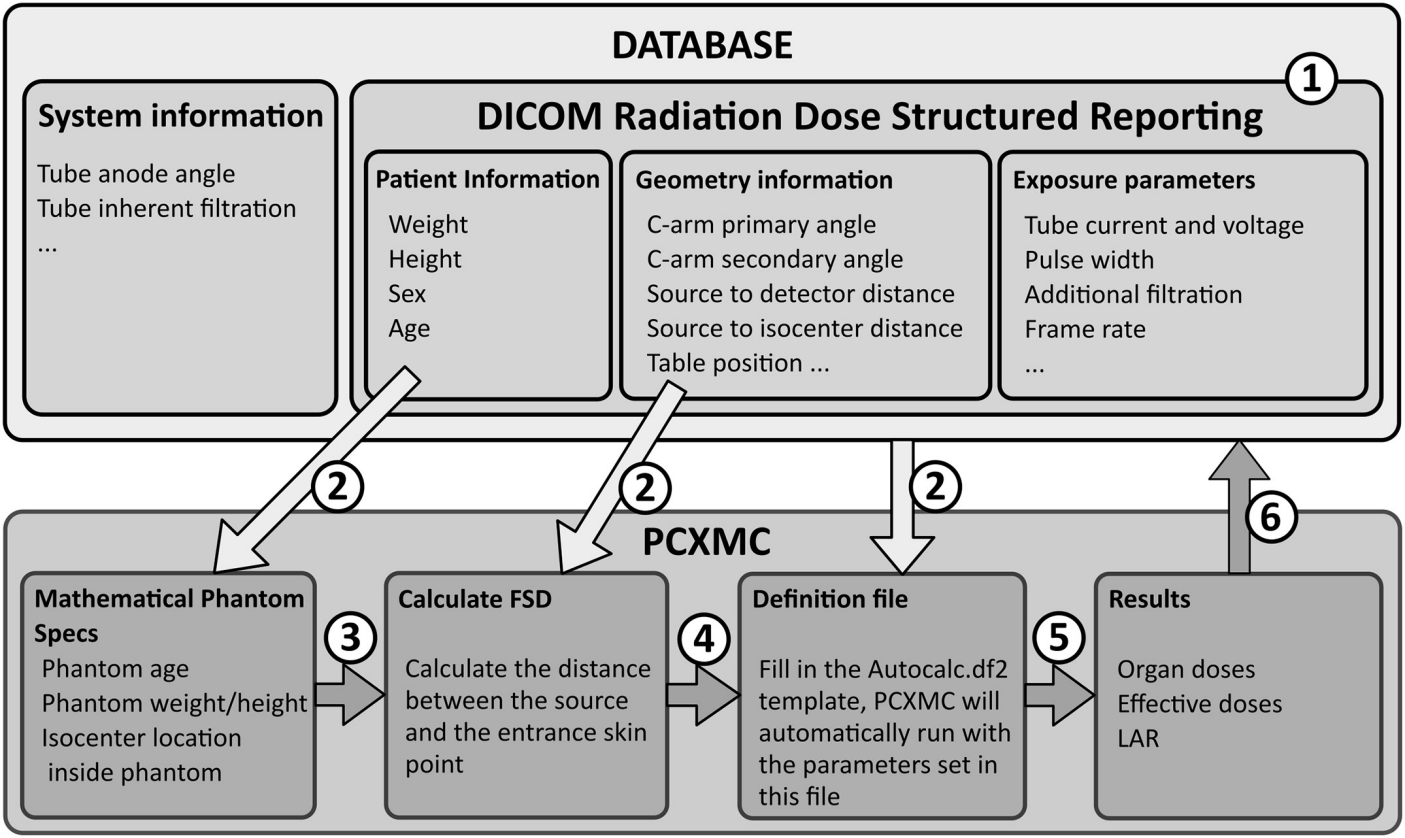
Automated risk assessment dataflow. 1: DICOM RDSR data are stored in Database. 2: Database is queried for irradiation events. 3: Phantom specifications are determined by patient age, height and weight. 4: FSD is calculated and stored with other data of the queried irradiation event into a definition file. 5: Definition file is simulated by executing PCXMC. 6: Results are stored back into the database.

Organ and effective doses are calculated using PCXMC, a Monte Carlo simulations software using mathematical phantoms [15]. The Monte Carlo method simulates photon transport through the phantom according to the stochastic nature of the different physical interaction processes between photons and matter. For each interaction the energy deposition and therefore dose is calculated taking into account the tissue characteristics of the organ the photon interacted with. The 222 procedures included in this study account for 14,013 irradation events. Each of these exposures are simulated using PCXMC. Due to the vast amount of irradiation events, the simulations were automated using in-house developed scripts and a customized database. Fig 1 gives a schematic overview of this process. First, RDSR data at the end of each examination are forwarded from DoseWatch to a customized database. Second, this database is then queried for the irradiation event data of the procedure. Third, a mathematical phantom is selected based on patient age and is adjusted to the patient’s height and weight. Assuming that the heart of the patient is positioned at the isocenter of the C-arms, the focal spot to entrance skin point distance (FSD) can be calculated from the previously determined phantom specifications and C-arm position. Fourth, the FSD and the rest of the irradiation event data are filled into the Autocalc definition file. Fifth, as soon as PCXMC is executed, the Autocalc definition file will be simulated. Sixth, lifetime attributable risks (LARs) are calculated from the organ doses and are finally stored together with the organ and effective doses into the database. The process is repeated for each irradiation event.

Conversion factors for organ doses (CF_OD_) and effective dose (CF_ED_) from DAP will be calculated based on the results obtained by the process described above. Conversion factors are divided into the weight categories recommended by the PiDRL project.

### Risk estimation

Lifetime attributable risk (LAR) of cancer incidence and mortality are determined according to the BEIR VII Phase 2 risk models. Risk models are available for several types of cancers and take into account the age- and gender-dependent incidence and mortality rates within the Euro-American population. The LAR factors of tables 12D-1 and 12D-2 of the BEIR VII Phase 2 report are adjusted to a dose and dose rate effectiveness factor (DDREF) of 2, as recommended by the ICRP [16].

DAP to LAR per 100,000 incidences or deaths conversion can be estimated by first applying the aforementioned DAP to organ dose conversion factors and subsequently using tables 12D-1 and 12D-2 of the BEIR VII Phase 2 report.

### Statistical analysis

For significance testing of DAP and normalized DAP differences between procedure groups, a Kruskal Wallis H test was performed. Subsequently, pairwise comparisons were performed using Dunn’s procedure with a Bonferroni correction for multiple comparisons.

Correlation between DAP and body weight and DAP and the product of body weight and fluoroscopy time was assessed via linear regression. Conversion factors from DAP to organ and effective dose, are determined as the slope coefficient of a linear regression analysis.

All statistical analyses were performed using IBM SPSS Statistics 25 (IBM corp, USA).

## Results

### Diagnostic reference levels

Taking into account all procedures, the DAP and DAP_BW_ values range from 16 to 17844 cGy.cm^2^ and from 0.3 to 254.9 cGy.cm^2^/kg respectively. Fluoroscopy times are between 0.5 and 100.4 minutes and the number of cinegraphic frames varied from 0 to 2162 frames. Additionally the air kerma, measured at the interventional reference point, is between 0.4 and 1454.7 mGy. Median contribution of fluoroscopy to total DAP for all interventional procedures is 91%, compared to 77% for diagnostic procedures. The latter difference was statistically significant. Furthermore, the biplane configuration is applied in 73% of interventional procedures and 85% of the diagnostic procedures. Table 1 provides the data in more detail, for different types of catheterization procedures, as median (P25-P75) values with P25 and P75 the 25^th^ and 75^th^ percentile respectively. The primary DRL values (75^th^ percentiles) are given in Table 2, for the two potential DRL parameters, i.e. DAP_BW_ and DAP_BWxFT_.

**Table 1:** Radiation exposure parameters per procedure type.

	DAP	Air kerma	Fluoroscopy time	Number of cinegraphic frames	Effective dose (ICRP_103_)
	[cGy.cm^2^]	[mGy]	[s]	[–]	[mSv]
**All (n = 222)**	193.67 (68.48–709.93)	27.05 (11.52–92.27)	477.50 (281–1088)	253 (96–578)	1.83 (0.91–5.36)
**Diagnostic (n = 33)**	200.31 (58.38–1187.179)	26.43 (11.21–114.73)	481 (267–1110)	456 (319–908)	3.34 (1.32–6.5)
**Interventional (n = 189)**	189.29 (68.93–694.81)	27.17 (12.20–90.60)	476 (296–1068)	226 (17–532)	1.60 (0.90–5.20)
**ASD closure (n = 33)**	135.2 (71.67–325.19)	13.88 (7.94–27.5)	254 (185–436)	0 (0–11)	0.78 (0.55–1.66)
**PFO closure (n = 17)**	355.35 (234.11–450.04)	26.93 (14.04–34.55)	243 (168–276)	0 (0–10)	0.9 (0.53–1.04)
**PDA closure (n = 18)**	87.35 (49.83–117.09)	15.03 (10.02–21.41)	358.50 (275–419)	172 (132–233)	1.22 (0.95–1.58)
**Single Balloon Valvuloplasty (n = 43)**	92.6 (45.17–226.38)	15.45 (10.47–39.33)	490 (408–851.50)	216 (125–294)	1.49 (0.92–3.14)
**Single Balloon Angioplasty (n = 34)**	118.75 (51.22–414.31)	23.25 (12.35–84.44)	486.50 (311–964)	324 (234–598)	2.06 (1.19–6.06)
**Multi Balloon Dilatation (n = 13)**	736.19 (254.53–1255.07)	75.91 (48.17–210.24)	1142 (729–1584)	452 (308–890)	4.03 (3.2–8.84)
**Pulmonary Valve Replacement (n = 7)**	9335.03 (7664.79–12212.58)	923.93 (776.39–1008.7)	2055 (1745–3566)	1274 (1210.5–1797.5)	32.84 (30.94–36.29)
**Stenting (n = 14)**	1957.23 (694.81–3906.06)	176.18 (102.85–434.30)	1642 (977–1870)	1052 (719–1356)	10.59 (6.84–12.38)

Cumulative procedure parameter values indicated by the X-ray modality and the resulting effective dose. Values are presented as median (P25-P75); ASD: atrial septal defect; PDA: patent ductus arteriosus; PFO: patent foramen ovale.

**Table 2:** DRL parameters per procedure type.

	DAP_BW_ [cGy.cm^2^.kg^-1^]	DAP_BW x FT_ [cGy.cm^2^.kg^-1^.min^-1^]
**All (n = 222)**	30.30	1.99
**Diagnostic (n = 33)**	33.54	3.59
**Interventional (n = 189)**	29.94	1.74
**ASD closure (n = 33)**	10.72	1.48
**PFO closure (n = 17)**	5.69	1.45
**PDA closure (n = 18)**	9.01	1.33
**Single Balloon Valvuloplasty (n = 43)**	19.21	1.53
**Single Balloon Angioplasty (n = 34)**	41.99	2.01
**Multi Balloon Dilatation (n = 13)**	55.99	1.91
**Pulmonary Valve Replacement (n = 7)**	153.32	3.50
**Stenting (n = 14)**	71.89	3.21

Primary DRL parameters. 75^th^ percentiles of the distribution per procedure group are given; ASD: atrial septal defect; PDA: patent ductus arteriosus; PFO: patent foramen ovale.

For both parameters DAP_BW_ and DAP_BWxFT_, differences between procedure types were statistically significant (p<0.0005). Considering DAP_BW_, pairwise comparison showed a statistically significant difference for 15 out of 36 possible pairs. For DAP_BWxFT_, only six pairs showed a statistically significant difference.

Linear regression between DAP and body weight results in an R^2^ value of 0.39 for all procedures combined. Table 3 documents median (P25-P75) values of DAP in each weight category suggested by RP 185 and one additional weight category larger than or equal to 80 kg. The R^2^ values for the separate procedure types range from 0.35 to 0.88. Linear regression analysis between DAP and the product of body weight and fluoroscopy time yields an R^2^ of 0.86 for all categories combined. When grouping the procedures in various procedure types, R^2^ values ranged from 0.73 to 0.98.

**Table 3:** DAP per weight category.

DAP [Gy.cm^2^]	Weight category [kg]					
	< 5	5 − < 15	15 − < 30	30 − < 50	50 − < 80	≥ 80
**n**	25	72	43	18	52	12
**Median**	0.62	0.73	1.87	7.12	8.68	31.84
**P25**	0.38	0.41	0.82	2.77	3.79	5.12
**P75**	1.15	1.35	4.40	24.74	37.14	75.25

DAP per weight category, for all procedure types combined.

### Organ and effective doses

Only the organ doses relevant to calculate lifetime risk are taken into account in this study, including the heart dose. Apart from the heart, the highest organ doses relevant for LAR calculation, i.e. for liver, lungs, breasts, remainder tissue and active bone marrow are provided as median (P25-P75) values in Table 4.

**Table 4:** Organ doses per procedure type.

	Heart	Liver	Lungs	Breasts	Remainder	Active bone marrow
	[mGy]	[mGy]	[mGy]	[mGy]	[mGy]	[mGy]
**All**	5.26 (2.86–17.56)	1.36 (0.6–4.37)	5.84 (2.91–19.52)	3.31 (1.31–8.88)	1.28 (0.67–4.13)	1.24 (0.5–3.56)
**Diagnostic**	8.57 (3.37–21.97)	2.89 (0.79–5.85)	11.2 (4.11–23.49)	6.01 (2.93–11.13)	2.3 (0.87–4.68)	1.48 (0.59–4.28)
**Interventional**	4.94 (2.82–16.55)	1.17 (0.59–4.21)	5.32 (2.91–18.54)	2.94 (1.28–8.38)	1.12 (0.66–3.55)	1.17 (0.5–3.35)
**ASD closure**	2.40 (1.33–4.03)	0.56 (0.33–1.22)	2.75 (1.85–5.01)	1.05 (0.68–2.19)	0.7 (0.37–1.4)	0.57 (0.35–1.45)
**PFO closure**	2.95 (1.69–3.74)	0.57 (0.26–0.66)	3.01 (1.97–3.43)	0.6 (0.36–0.78)	0.8 (0.39–0.88)	1.27 (0.79–1.54)
**PDA closure**	3.75 (3.13–5.32)	0.83 (0.59–1.04)	4.02 (2.89–5.86)	3.08 (1.84–4.34)	0.77 (0.62–1.02)	0.5 (0.39–0.62)
**Single Balloon Valvuloplasty**	4.65 (3.03–9.86)	0.99 (0.7–2.35)	4.98 (3.09–9.35)	2.67 (1.84–5.8)	1.01 (0.63–2.15)	0.8 (0.42–1.88)
**Single Balloon Angioplasty**	6.23 (3.66–18.53)	1.64 (0.72–5.15)	5.84 (3.92–21.73)	3.91 (1.77–7)	1.39 (0.78–4.36)	0.79 (0.42–3.35)
**Multi Balloon Dilatation**	13.46 (8.9–34.46)	3.33 (2.51–8.08)	15.47 (11.04–43.72)	3.93 (2.66–14.07)	2.57 (2.08–7.99)	3.43 (2.36–6.96)
**Pulmonary Valve Replacement**	110.17 (106.98–123.98)	26.61 (24.5–34.06)	114.87 (109.13–136.37)	64.5 (31.47–66.27)	22.59 (18.54–28.54)	26.89 (22.24–39.02)
**Stenting**	29.84 (22–47.16)	7.37 (6.05–11.65)	39.24 (24.32–53.72)	11.83 (8.28–16.87)	7.11 (4.34–11.16)	7.77 (4.02–9.72)

Heart dose and the top five organ doses to organs accounting for LAR determination; ASD: atrial septal defect; PDA: patent ductus arteriosus; PFO: patent foramen ovale.

The effective doses are calculated using the tissue weighting factors of ICRP publication 103 (ED_103_) and are tabulated in Table 1 for several procedure types. ED_103_ ranges from 0.05 to 61.68 mSv with an average value of 5.1 mSv.

A strong correlation between DAP and ED_103_ (CF_ED_) is noticed (R^2^ = 0.85), which is improved when the data is grouped into the weight groups recommended by the RP 185 (R^2^ from 0.95 to 0.97). The correlation is even better when considering DAP_BW_ instead of DAP for each weight group (R^2^ from 0.96 to 0.99). Conversion factors are also calculated for the frontal and lateral C-arm separately. The results of linear regression analysis between DAP_BW_ and ED_103_, i.e. the slope coefficients and limits of the 95% confidence intervals, are shown in Table 5. All linear regressions are statistically significant. Since the X-ray modality is a biplane modality and DICOM RDSR communicates cumulative DAP values for the whole procedure for each C-arm, we also provide separate conversion factors for the frontal and lateral C-arm. The sum of the effective doses calculated for both C-arms using their corresponding CF_ED_, yields the cumulative effective dose of the procedure. The lateral C-arm in current study almost exclusively acquires lateral projections (on average LAO87°-CRA1°). Hence, when lateral acquisitions are not employed on a monoplane system or during a monoplane procedure, the Frontal CF_ED_ from Table 5 can also be used as a single conversion factor to calculate ED from DAP. When the system only reports one cumulative DAP value per procedure and lateral acquisitions are applied, we suggest to use the Total CF_ED_.

**Table 5:** CF_ED_ per weight category.

CF_ED_ [mSv.Gy^-1^.cm^-2^.kg]		Weight category [kg]						
		< 5	5 − < 15	15 − < 30	30 − < 50	50 − < 80	≥ 80	All
**Total**	**n**	25	72	43	18	52	12	222
	**Slope**	**14.17**	**14.07**	**19.79**	**20.63**	**20.89**	**25.59**	**19.84**
	**LL**	13.50	13.48	18.60	19.38	19.67	23.35	19.17
	**UL**	14.84	14.66	20.98	21.88	22.10	27.83	20.51
**Frontal**	**n**	25	72	43	18	52	12	222
	**Slope**	**11.37**	**11.52**	**15.32**	**16.71**	**16.17**	**19.42**	**15.62**
	**LL**	11.04	11.09	14.87	15.99	15.31	18.09	15.17
	**UL**	11.69	12.96	15.76	17.43	17.03	20.76	16.07
**Lateral**	**n**	25	64	28	14	26	8	165
	**Slope**	**18.29**	**18.65**	**25.22**	**26.28**	**31.58**	**35.97**	**27.81**
	**LL**	17.63	17.73	23.61	23.70	30.12	33.47	26.74
	**UL**	18.96	19.57	26.82	28.87	33.03	38.46	28.89

Results of the linear regression analysis between ED and DAP_BW_. The slope coefficients, i.e. the conversion factors, are shown in bold. Total CF_ED_ is used when one cumulative DAP value is available per procedure, Frontal and Lateral CF_ED_ are used for each C-arm separately on a biplane system; LL: lower limit of the 95% confidence interval; UL: upper limit of the 95% confidence interval; n: number of values included in the analysis.

Likewise conversion factors from DAP_BW_ to OD are reported instead of DAP to OD, since the correlations were better and 95% confidence intervals were smaller, when normalizing DAP by patient weight. The conversion factors are tabulated in Table 6.

**Table 6:** CF_OD_ per weight category.

CF_OD_ [mGy.Gy^-1^.cm^-2^.kg]		Weight category [kg]						
		< 5	5 − < 15	15 − < 30	30 − < 50	50 − < 80	≥ 80	All
**Heart**	**Biplane**	37.38	37.73	68.87	63.08	65.17	91.26	62.07
	**Frontal**	26.17	24.76	41.88	32.14	44.28	68.01	41.73
	**Lateral**	54.48	60.78	99.92	131.00	110.94	127.90	101.91
**Stomach**	**Biplane**	7.09	7.93	10.68	9.76	8.42	11.11	8.77
	**Frontal**	6.29	7.81	9.26	10.98	8.65	12.31	8.99
	**Lateral**	7.98	7.91	12.77	7.76	7.14	8.78	8.12
**Colon**	**Biplane**	1.10	1.06	0.87	0.68	0.50	0.77	0.68
	**Frontal**	0.90	0.88	0.76	0.61	0.40	0.54	0.53
	**Lateral**	1.40	1.39	1.04	0.77	0.72	1.14	0.97
**Liver**	**Biplane**	11.93	12.77	14.92	19.90	18.29	23.16	17.57
	**Frontal**	7.65	8.54	10.33	15.65	12.36	11.00	11.71
	**Lateral**	17.72	21.01	20.96	24.60	32.01	42.32	28.53
**Lungs**	**Biplane**	41.73	45.20	76.48	77.12	75.93	95.01	71.34
	**Frontal**	36.99	39.47	53.92	61.82	56.02	60.19	53.66
	**Lateral**	49.10	54.99	103.51	109.44	120.27	148.40	104.98
**Urinary Bladder**	**Biplane**	0.31	0.23	0.11	0.06	0.03	0.06	0.09
	**Frontal**	0.26	0.19	0.10	0.05	0.02	0.03	0.06
	**Lateral**	0.39	0.32	0.14	0.08	0.05	0.09	0.14
**Thyroid**	**Biplane**	3.57	3.33	3.02	2.17	1.69	2.40	2.24
	**Frontal**	2.72	2.69	2.10	2.17	1.35	1.60	1.72
	**Lateral**	4.77	4.68	4.29	2.36	2.39	3.77	3.29
**Active Bone Marrow**	**Biplane**	5.56	6.45	9.92	13.94	21.25	26.37	17.37
	**Frontal**	6.47	7.36	9.49	16.43	23.13	30.76	19.76
	**Lateral**	4.31	4.66	10.92	8.92	15.16	19.15	12.03
**Breasts**	**Biplane**	34.72	27.65	27.33	31.92	27.13	27.25	28.23
	**Frontal**	21.37	15.40	22.73	13.39	9.86	9.80	11.98
	**Lateral**	54.60	50.87	31.47	51.05	72.57	62.56	60.24
**Oesophagus**	**Biplane**	32.94	31.23	45.22	40.50	30.97	62.48	31.83
	**Frontal**	26.71	27.24	37.38	38.10	30.22	67.03	30.53
	**Lateral**	42.25	38.09	62.84	35.27	43.47	55.44	44.92
**Ovaries**	**Biplane**	0.67	0.56	0.34	0.19	0.12	0.21	0.25
	**Frontal**	0.56	0.46	0.29	0.17	0.08	0.12	0.18
	**Lateral**	0.85	0.74	0.42	0.24	0.20	0.36	0.38
**Remainder Male**	**Biplane**	9.32	10.53	14.36	15.31	14.50	19.51	14.17
	**Frontal**	7.94	9.57	11.94	14.31	12.78	17.58	12.65
	**Lateral**	11.10	11.83	17.50	18.21	18.10	22.45	17.05
**Remainder Female**	**Biplane**	9.34	10.54	14.37	15.32	14.50	19.52	14.18
	**Frontal**	7.95	9.58	11.95	14.32	12.79	17.59	12.66
	**Lateral**	11.12	11.85	17.51	18.22	18.10	22.46	17.06

Results of the linear regression analysis between OD and DAP_BW_

### Risk estimation

Lifetime attributable risk is calculated according to the BEIR VII Phase 2 risk models. The results are tabulated in Table 7 for all cancers combined as median(P25-P75) values per 100,000 incidences or deaths. Including all procedures, the median lifetime risk for cancer incidence and mortality are respectively equal to 0.034% and 0.021% with respective ranges from 0.001% to 0.315% and from 0.001% to 0.146%.

**Table 7:** Incidence and mortality LAR values.

LAR	Male		Female	
[per 100,000]	Incidence	Mortality	Incidence	Mortality
**All procedures**	(n = 116)		(n = 106)	
**All cancers**	23 (11–57)	17 (8–43)	55 (25–129)	30 (15–90)

Median (P25-P75) LAR (BEIR) values per 100,000 incidences or deaths

Using the conversion factors in Table 6, one can calculate the organ dose from normalized DAP values and subsequently determine the corresponding LAR using tables 12D-1 and 12D-2 of the BEIR VII Phase 2 report. As a practical example for this workflow, LAR for lung cancer incidence is calculated here from DAP by the given conversion factors: For a 10-year-old male patient weighing 35 kg, a DAP equal to 683.13 cGy.cm^2^ was noted, i.e. a DAP_BW_ equal to 683.13 / 35 = 19.52 cGy.cm^2^.kg^-1^. From Table 4 we get a combined biplane conversion factor to lung dose equal to 77.117 mGy.Gy^-1^.cm^-2^.kg. Multiplying this factor with the DAP_BW_ previously determined, gives us a lung dose of 77.117×19.52/100 = 15.05 mGy. More accurate organ dose estimates will be obtained when applying the frontal and lateral C-arm conversion factors separately on their respective DAP contribution and then adding both results to get the total organ dose. Table 12D-1 of the BEIR VII Phase 2 report shows the estimated lifetime risk of being diagnosed with lung cancer for a male exposed to 0.1 Gy at age 10 as 216 per 100,000, which is adjusted by a DDREF of 1.5. Our example patient received 15.05 mGy of dose to the lungs, which results in an estimate of 0.01505/0.1×216 = 33 per 100,000. When adjusting for a DDREF of 2, instead of the tabulated 1.5, we obtain an estimate of 33×1.5/2 = 25 per 100,000.

## Discussion

### Diagnostic reference levels

Median DAP values are amongst the lowest values found in literature for patient weight lower than 30 kg. However, 75^th^ percentile values are in the higher range of values compared to literature. This is due to the relatively low number of procedures in this study, the high differences in procedure complexity and the higher variability noticed for current study in the highest weight category.

Onnasch et al., Chida et al. and Ubeda et al. all found reasonable correlations between DAP and body weight [10, 11, 17]. Ubeda et al. reported R^2^ values between 0.247 and 0.698 for different types of procedures, which is confirmed in the current study with values between 0.354 and 0.883 [11]. Therefore DAP_BW_ is suggested here as a primary DRL parameter for different procedure types, with respective median (P25-P75) values of 0.163 (0.088–0.324) and 0.096 (0.051–0.294) Gy.cm^2^/kg for diagnostic and interventional procedures. These DRLs, as wel as the median values, are lower than the ones reported by Onnasch et al., i.e. 0.409 and 0.559 Gy.cm^2^/kg for diagnostic and therapeutic procedures respectively [17]. The DRLs are higher than the ones reported by Ubeda et al, i.e. 0.163 and 0.170 Gy.cm^2^/kg for diagnostic and therapeutic procedures respectively, although the median values of both studies are matching quite well [11].

In contrast to literature, current study recorded higher median DAP for diagnostic than for interventional procedures. This can be attributed to the extensive use of transesophageal ultrasound, during ASD, PFO and VSD procedures in our department. Hence, total DAP of these procedures is almost entirely attributed to fluoroscopy, resulting in low DAP values. ASD, PFO and VSD procedures account for 29% of all interventional procedures. Furthermore, biplane configuration was used more frequently in diagnostic procedures. Additionally, the number of frames is almost doubled in diagnostic procedures compared to interventional procedures, while fluoroscopy time was equal in both groups, as shown in Table 1. Thus, cinegraphy is more frequently used in diagnostic examinations. This is as expected since during diagnostic examinations the cardiologists want to have a good understanding of the morphology and pressure values of a larger anatomic region, while for interventional procedures they focus on a specific feature or malformation (e.g. stenosis of the pulmonary artery or valve, aortic coarctation, …) of the anatomic area.

RP 185 states that DRLs should be based on statistically relevant numbers of patient dose data. They recommend to use at least 20 cases per procedure type and per patient group for complex procedures such as fluoroscopically guided procedures. Yet, Table 1 shows several procedure types with less than 20 cases, after 21 months of data collection. The DRLs from the latter procedure types should therefore be consulted with care since they might be less meaningful. However, smaller departments inherently deal with too few data. Hence with DAP_BW_, it will probably not always be possible to set DRL values, or to gather sufficient data to compare with existing DRLs, for all procedure types in smaller departments within a reasonable time frame.

With the nine different procedure types in this study, 36 pairwise procedure type comparisons can be made. As suggested by the European guidelines, separate local DRLs were proposed for each procedure type. This suggestion is validated in this study for DAP_BW_, since 15 out of 36 (i.e. 42%) possible pairwise procedure comparisons, subsequent to the Kruskal Wallis H test, were statistically significant. By splitting the procedures into different procedure types, one thus takes into account inter-procedure type differences in complexity. However, intra-procedure type differences are not considered in this way. When normalizing DAP by the product of body weight and fluoroscopy time, i.e. DAP_BWxFT_, only 6 out of 36 pairwise procedure comparisons showed a statistically significant difference. Additionally, in each of the latter six comparisons, one of the procedure pairs were either stenting or pulmonary valve replacement procedures, which both had significant outliers and which were procedures with respectively the lowest and third lowest number of cases. Hence we can hypothesize that DAP_BWxFT_ inherently takes into account inter-procedure type differences.

A strong linear correlation (R^2^ = 0.88) between DAP and the product of body weight and fluoroscopy time for all procedures combined was observed. Taylor et al. determined optimal sample sizes to calculate DRL values for CT, based on the 95% confidence interval in percentage of their median (CI95/med) being below 10% [18]. Considering all procedures, CI95/med values in current study were 63.5, 51.2 and 16.7% for DAP, DAP_BW_ and DAP_BWxFT_ respectively. This trend remains when considering the procedure types separately. Thus, applying DAP_BWxFT_ also seems to account for intra-procedure complexity differences and yields narrower confidence intervals even when all procedures are pooled into one group.

Chida et al. and Nguyen et al. also observed better correlation between DAP and BWxFT, than between DAP and body weight only [10, 19]. Sullivan et al. reported data of procedures performed on the same biplane X-ray modality as in this study. They observed an average (95% CI) DAP_BWxFT_ of 2.18 (2.02–2.34) cGy.cm^2^.kg^-1^.min^-1^, agreeing closely with our value of 1.80 (1.65–1.96) cGy.cm^2^.kg^-1^.min^-1^ [20].

These findings suggest that, when considering DAP_BWxFT_ as the primary DRL parameter, only one DRL value for all procedures combined is feasible. This is highly favorable for the many smaller paediatric catheterization departments with insufficient data to calculate DRL values per procedure type and body weight group in a reasonable time span, since the latter parameter seems to take into account both intra- and inter-procedure type differences. Whenever a high patient dose is reached, comparison to the DRL may help in finding the cause of said high exposure. For example, if a procedure’s DAP_BWxFT_ exceeds the DRL, several reasons may apply. Maybe the cinegraphy contribution was higher than usual, due to an increased number of acquisitions, or a high dose cinegraphy protocol was used. Selecting a wrong fluoroscopy protocol, with low filtration and therefore higher dose, could be the cause. For small patients, anti-scatter grid may be inserted, causing higher exposure than usual. This shows that secondary DRL parameters, like fluoroscopy time and number of frames, can assist the primary DRL in this root analysis. Most dose management systems currently available provide alerting and reporting tools. At the end of the procedure, data are sent to the dose management software, which will compare data of the current study to the DRL and/or to statistics calculated from the previous procedures. If the dose of the current procedure exceeds the aforementioned values, the health professional can be notified by an alert popping up in a worklist, or by e-mail. Additionally, these systems can send monthly reports, showing a list of the procedures where the doses were unusually high.

### Effective doses, organ doses and risk estimation

Effective dose in current study ranges from 0.05 to 61.68 mSv with respective median(P25-P75) values of 3.34 (1.32–6.5) and 1.58 (0.86–4.74) mSv for diagnostic and interventional procedures. Bacher et al. calculated effective dose using detailed Monte Carlo simulations [6]. They calculated a median effective dose equal to 4.6 and 6.0 mSv for diagnostic and therapeutic catheterizations respectively. Barnaoui et al. used conversion factors from literature to estimate effective dose and found a mean value of 4.8 and 7.3 mSv for diagnostic and therapeutic procedures respectively. Johnson et al. calculated effective dose from measurements performed in anthropomorphic phantoms using TLD dosimeters. Diagnostic procedures resulted in a median effective dose of 9.1 mSv, while therapeutic procedures yield 13.77 mSv [14]. Effective doses determined in the current study are amongst the lowest values found in literature, as shown in Table 6 from Ubeda et al. [21]. This table shows only lower values reported by Ubeda et al. For patient weight <30 kg and >60 kg we see about the same median effective doses, but for weights between 30 and 60 kg the effective dose is higher in this study. This can be attributed to a limited patient cohort in the latter weight category and a different weight distribution since CF_ED_ values are higher in this study for patients <30 kg and smaller for patients >50 kg. Furthermore Ubeda et al. only included patients younger than 16 years of age. Current study did not exclude any congenital heart disease patients based on patient age, with median (P25-P75) age equal to 4.3 (0.9–15.6) years. The youngest patient was 2 days old, the oldest one was 69.6 years [21].

A summary of conversion factors from DAP to effective dose (CF_ED_) are shown in Table 8, which is an adjustment of Table 4 of Harbron et al. [13]. Effective dose conversion factors using tissue weighting factors of ICRP publication 60 are also provided in the latter table for comparison with literature. Conversion factors of the current study are closest to the values of Barnaoui et al. If we would have calculated effective doses according to the latter conversion factors, we would have overestimated and underestimated effective dose by on average 14.3% and 14.5% for frontal and lateral exposures respectively [9]. Applying conversion factors from Ubeda et al. to the procedures of current study would have yielded on average an underestimation by 28% [21].

**Table 8:** CF_ED_ in literature.

CF_ED_ [mSv.Gy^-1^.cm^-2^]	Barnaoui	Schmidt	Onnasch	Karambatsakidou	Current study	Current study
	ICRP_103_	ICRP_60_	ICRP_60_	ICRP_60_	ICRP_103_	ICRP_60_
**Age**	**AP/LAT**	**AP/LAT**	**AP+LAT**	**AP/AP+LAT**	**AP/LAT/AP+LAT**	**AP/LAT/AP+LAT**
**0 y** **(3.4 kg)**	3.5/3.5	2.05/2.34	2.72	3.7/3.7	2.26/4.83/3.3	1.94/3.74/2.68
**1 y** **(9.2 kg)**	1.6/2.6	0.82/1.16	1.01	1.9/1.9	1.35/2.52/1.71	1.22/1.98/1.46
**5 y** **(19 kg)**	0.8/1.3	0.42/0.64	0.49	1.0/1.0	0.89/1.48/1.18	0.8/1.33/1.06
**10 y** **(32.4 kg)**	0.5/0.8	0.24/0.38	0.29	0.6/0.7	0.46/0.79/0.59	0.45/0.66/0.53
**15 y** **(56.3 kg)**	0.3/0.4	0.13/0.22	0.16	0.4/0.4	0.33/0.5/0.42	0.32/0.4/0.36

Comparison to literature, adjusted from Harbron et al.

Median (P25-P75) lifetime risk for cancer incidence and mortality from this study is respectively equal to 0.034 (0.014–0.103)% and 0.021 (0.009–0.063)%. Few studies in literature included LAR calculations. Bacher et al. showed a median LAR for stochastic effects of 0.08% for all patients combined. For patients younger than 1 year of age, their results show a median LAR of 0.10%, this study reports a LAR of 0.044%. For age groups 2–5 and 6–10 years they note a LAR of 0.08% and 0.05% respectively, according to the current study these values are 0.029% and 0.030% for the same respective age groups [6]. Johnson et al. report an overall median LAR of cancer incidence of 0.065%, however their study only included children younger than or equal to 6 years. For the same age groups we report a LAR of 0.034%. To be able to compare with LAR values from literature, the LAR values from current study are reported only in this paragraph for a DDREF of 1.5.

The use of ED and LAR has some inherent limitations. ED was not intended to assess stochastic risk and should not be used for individual risk assessment. The ED calculated in current study can for example be used to compare similar procedures in other centres working with either similar or different technologies, as also suggested by ICRP_103_. Appropriate use of effective dose is discussed in detail by Fisher et al [16, 22]. The BEIR committee states that due to limitations in the data used to develop their risk models for low levels of low-LET ionizing radiation, risk estimates are uncertain, and estimates that are a factor of two or three larger or smaller cannot be excluded [5]. Additionally, when calculating individual risk, we are not assessing risk to one actual individual, we are assessing the probability of a biological effect (e.g. cancer incidence or mortality) in a theoretical population with the same characteristics as the actual patient (e.g. age and sex) and that one theoretical individual in the latter population might exhibit this biological effect [23]. Communicating risk to an individual patient should therefore be discouraged since the actual risk for one specific patient might deviate significantly from the calculated LAR. The latter parameter can be more meaningful on a patient population level, where it can for example be used to compare different technologies for similar procedures [24, 25]. The only parameter obtained through the Monte Carlo calculations that could be communicated to one specific patient is organ dose. However we should note that an actual patient is still different from the age and size adjustable hermaphrodite mathematical phantoms as implemented in PCXMC.

### Automated risk assessment

Brambilla et al. was also able to calculate organ and effective doses in coronary angiography and PCI procedures, taking into account each fluoroscopy and cinegraphy exposure per procedure. However, their simulations were only performed on an adult mathematical phantom, including 2 isocenter positions, one for male and one for female. This study adjusts the mathematical phantom dimensions and isocenter positions according to the patients age and size, for each irradiation event, yielding a more accurate resemblance to reality [26].

Using in-house developed scripts, the Monte Carlo simulation results are stored together with the exposure parameters of the RDSR into one database. Integrating this workflow into dose management software, could expand their scope to organ dose and risk management. Making risk information centrally available for users, could help selecting the optimal medical imaging exam and device when multiple options are available and could improve risk communication towards the patient, the general public and other physicians. Future epidemiology research can benefit from the availability of patient-specific organ doses in dose management systems, as implemented in current study.

## Conclusion

The average and median values of DAP_BW_ and DAP_BWxFT_ analyzed in this study are well matching the values found in literature. DAP_BWxFT_ is the recommended primary DRL parameter, since linear regression between DAP and BWxFT showed the best correlation combined with a narrower 95% confidence interval. When sufficient data are available, DRLs should be calculated for separate procedure types. However, pooling procedures into one single group, still yields appropriate DRL values for DAP_BWxFT_, which is recommended in smaller departments with less data.

When Monte Carlo simulations are not an option, DAP_BW_ can serve as an excellent indicator of organ and effective doses. We recommend to use different conversion factors for different body weight groups when Monte Carlo simulations are no option. Furthermore, using different conversion factors for frontal and lateral projections would further improve accuracy of organ and effective doses. However, conversion factors in literature should be chosen with much care, trying to match exposure parameters and patient size parameters as much as possible to your own practice and patient population.

## Supporting information

S1 Dataset An anonymous dataset containing the exposure information of any irradiation event available in the RDSRs of all included procedures. (XLSX)

## Abbreviations

ASD: Atrial septal defect
BWxFT: Product of body weight and fluoroscopy time
CF_ED_: Conversion factor from DAP to effective dose
CF_OD_: Conversion factor from DAP to organ dose
CI95/med: 95% confidence Interval as percentage of the median value
DAP: Dose area product
DAP_BW_: Dose area product normalized by body weight
DAP_BWxFT_: Dose area product normalized by the product of body weight and fluoroscopy time
DDREF: Dose and dose rate effectiveness factor
DRL: Diagnostic reference level
ED_103_: Effective dose calculated acoording to the tissue weighting factors from ICRP publication 103
ED_60_: Effective dose calculated acoording to the tissue weighting factors from ICRP publication 60
ESR: European Society of Radiology
FSD: Focal sport to entrance skin point distance
ICRP_103_: ICRP publication 103
ICRP_60_: ICRP publication 60
LAR: Lifetime attributable risk
OD: Organ dose
P25: 25^th^ percentile
P75: 75^th^ percentile
PDA: Patent ductus arteriosus
PFO: Patent foramen ovale
PiDRL: European diagnostic reference levels for paediatric imaging
RDSR: DICOM radiation dose structured reporting
TLD: Thermoluminescent dosimeter
